# A Comparative Survey of Historical DNA Degradation From Vertebrate Museum Specimens: Unexpected Utility of Fluid‐Preserved Specimens

**DOI:** 10.1111/1755-0998.70206

**Published:** 2026-10-20

**Authors:** Alexander T. Salis, Nasrin Ali, Shiva Karmakar, Jasleen Kaur, Brian Tilston Smith, Christopher J. Raxworthy

**Affiliations:** ^1^ Division of Vertebrate Zoology American Museum of Natural History New York New York USA; ^2^ Science Research Mentoring Program, Department of Education American Museum of Natural History New York New York USA

**Keywords:** alcohol, cryo‐collection, dried‐skin, formalin, historical DNA, museomics, skeleton, voucher specimens

## Abstract

Advances in museomics have expanded from dry and skeletal specimens to now include fluid‐preserved material as a source of DNA. However, our understanding of patterns of DNA degradation across specimens and tissue types remains limited, hindering justification for targeting optimal DNA sources from museum voucher specimens. Here we quantify DNA quality across taxonomic groups (amphibians, squamates, and birds) and tissue types to benchmark DNA yield, fragmentation, endogenous content, and DNA damage in fluid‐preserved specimens versus more commonly used sources (frozen tissues, dried skins, and skeletons). Our tests showed that fluid‐preserved material had smaller fragments, decreased endogenous content, and increased deamination with age, indicative of extensive degradation over even short timescales. Unexpectedly, formalin‐fixed fluid‐preserved specimens did not show clear declines in DNA yield or fragment length with specimen age, suggesting formalin fixation initially fragments DNA but may partly decouple subsequent fragment‐length patterns from specimen age. DNA yield, fragment size, and endogenous content across tissue types in fluid‐preserved specimens showed both time‐ and tissue‐type dependent patterns. DNA from liver had the highest quality metrics in younger specimens but performed worse in the oldest specimens. In older specimens, the optimal tissue types included skin, muscle or bone across taxonomic groups. However, substantial variation between individual specimens of the same preparation type and age indicated that collection history also influences DNA degradation. Our comparative analyses reinforce earlier findings that fluid‐preserved specimens of all ages are viable DNA sources. We use these findings to refine DNA sampling strategies for incorporating fluid‐preserved voucher specimens into museomic research.

## 1 Introduction

Museomics—the generation of ‐omics data from natural history collections—has rapidly emerged as a major application of natural history collections (Blair 2024; Card et al. 2021; Fong et al. 2023; Raxworthy and Smith 2021). Traditional preservation methods vary widely between institutions and taxonomic groups, encompassing whole organisms (e.g., pinned insects and fluid‐preserved material) to partial voucher specimens such as skins, skeletons, pressed plant parts, and more recently tissues specifically preserved for biomolecular analyses (e.g., cryogenic biobank tissue collections). Frozen tissue collections, ideal for genetic research, have only emerged in the last 50 years, with dedicated tissue collection being established in the past few decades, thus limiting their temporal record (Edwards et al. 2005; Martin 2006; Radin 2015). Advances in DNA extraction techniques and the development of high‐throughput sequencing have broadened the scope of usable voucher specimens in genetic research and greatly increased the quantity of genetic data that can be obtained from natural history collections, spanning to whole genomes, population genomic data, and even epigenetic data (e.g., Burrell et al. 2015; Hahn et al. 2024; Hung et al. 2014; Hykin et al. 2015; Li et al. 2015; Smith et al. 2021; van der Valk et al. 2019).

DNA extracted from voucher specimens is often highly degraded—characterized by increased fragmentation and susceptibility to exogenous contamination—and is commonly referred to as historical DNA (hDNA; Billerman and Walsh 2019). Dried skins, mounts, and skeletal material have been among the most widely used sources of reliable hDNA (Caccone et al. 1999; Higuchi et al. 1984; Marsh et al. 2025; Mundy et al. 1997) and have consequently become routine sample types in museomic research (e.g., Blair et al. 2023; Ernst et al. 2022; Islam et al. 2024; Pacheco et al. 2022; Penna et al. 2024; Roycroft et al. 2021, 2022; Smith et al. 2021, 2020; Tsai et al. 2020). However, because there is a substantial taxonomic bias for vertebrates preserved with dried skins and mounts—largely mammals and birds—the groups where this preservation technique is not common have been vastly underrepresented in museomic research.

For some taxonomic groups (amphibians, non‐avian reptiles, and fishes) fluid preservation is the primary mode of specimen preparation, and for these groups museomic research has been less common. However, over the past decades substantial progress has been made in isolating endogenous DNA from fluid‐preserved specimens (Appleyard et al. 2021; Austin and Melville 2006; Bernstein and Ruane 2022; Brino et al. 2023; Derkarabetian et al. 2019; Hahn et al. 2022; Holleley and Hahn 2025; Hykin et al. 2015; McGuire et al. 2018; Pyron et al. 2022; Rancilhac et al. 2020; Ruane and Austin 2017; Scarsbrook et al. 2022; Straube et al. 2021; Stuart et al. 2006; Wood et al. 2018; Zacho et al. 2021). Success has been generally higher in specimens that were initially fixed with alcohol (not formalin), for younger formalin‐fixed specimens, and liver is considered to have a higher chance for successful DNA retrieval (Hahn et al. 2022; Hykin et al. 2015; O'Connell et al. 2022; Zacho et al. 2021). Failure rates likely remain high, and consistent trends that might help predict DNA extraction success across museum specimens have not yet been documented (Raxworthy and Smith 2021).

Prior to the 20th century, ethanol was the main fixative used to fix specimens (alcohol‐fixed), typically using 70%–75% ethanol (Simmons 2014). During the 20th century, fixing specimens with formalin became a common practice, typically using a 10% concentration of formalin solution that is in contact with the specimen for days to months before being transferred to ethanol for long term archiving. The presence of water in ethanol fixatives is likely to accelerate hydrolytic damage, especially if regular maintenance of specimens is not performed and ethanol concentration drops below 70% due to evaporation (Dabney et al. 2013; McGuire et al. 2018; Zacho et al. 2021). In formalin‐fixed specimens, this is further exacerbated through exposure to formalin, which has a high presence of water (~90%) and is known to cause miscoding lesions, crosslinks with DNA and protein, as well as potentially damaging and fragmenting the DNA directly (Do and Dobrovic 2015; Guyard et al. 2017; Hedegaard et al. 2014; Srinivasan et al. 2002; Watanabe et al. 2017; Williams et al. 1999). The damage and crosslinks caused by formalin‐fixation together with the often‐unknown fixation history of fluid‐preserved specimens are thought to remain the biggest impediments to fluid‐preserved specimens being used in museomic research (Holleley and Hahn 2025; Raxworthy and Smith 2021; Wandeler et al. 2007).

More generally, the available DNA in museum specimens (almost always less than 200 years old) has often experienced preservation regimes that differ from burial‐derived/natural contexts, including chemical fixation and preservation, chemical exposure from pest management practices, storage in fluids, and prolonged exposure to indoor museum environments. Although many damage processes (e.g., fragmentation, miscoding lesions, polymerase inhibition) also occur in burial/natural‐derived material such as Pleistocene bone, recent work has shown that museum storage environments and chemical treatments can impose substantial and sometimes accelerated DNA damage compared to if left in a natural context (Eriksen et al. 2025). These museum preservation strategies may alter DNA degradation pathways by inducing crosslinking, miscoding lesions, and accelerated fragmentation (Do and Dobrovic 2015; Hedegaard et al. 2014; Jensen et al. 2022; Srinivasan et al. 2002; Topfer et al. 2011; Williams et al. 1999). Despite these very different preservation histories, we are aware of no study that has explicitly compared the performance of different tissues in fluid‐preserved voucher specimens across preservation methods and time, and compared the results to other museum voucher specimens of differing preservation conditions. Because of this lack of comparative experimental data on historical DNA quality, we cannot yet be confident that researchers are targeting the optimal DNA sources from museum voucher specimens.

To fill this knowledge gap, in our study, we compared DNA extraction and sequencing measures across voucher types typical of vertebrates (amphibians, squamates, and birds) in natural history collections: (1) frozen tissues; (2) dried skins; (3) skeletons; and (4) fluid‐preserved specimens (both alcohol and formalin‐fixed) to test if different voucher types are associated with different DNA degradation profiles under archival conditions. As no broad comparisons of success across different tissue types had been performed, we also compared extraction and sequencing measures across skin, muscle, liver, and bone taken from the same alcohol or formalin‐fixed voucher specimen. We anticipated our results could help improve best practices for sampling both dry and fluid‐preserved material, thus increasing the utility of traditional museum voucher specimens in museomic research, and allowing the incorporation of additional rare specimens (potentially even extinct species) with minimal destruction to these precious resources.

## 2 Methods

### 2.1 Sample Acquisition

For vertebrate collections, DNA was extracted from four main types of specimens: (1) frozen tissue samples, (2) dried skins and mounts, (3) skeletons, and (4) fluid‐preserved specimens stored in ethanol (but initially fixed in either formalin or ethanol). We obtained 132 specimens from the American Museum of Natural History's Vertebrate Zoology collections. Our sampling scheme consisted of 70 avian specimens (dried skins = 42; formalin‐fixed fluid specimens = 22; and alcohol‐fixed fluid specimens = 6) and 62 herpetological specimens (dried skeletons = 10; formalin‐fixed fluid specimens = 29; and alcohol‐fixed fluid specimens = 23; Table 1). These specimens ranged in age from three to 152 years old, being collected between 1872 and 2021 and were grouped into young (collected after 1980) and old (collected before 1980) categories (see Table S1 for voucher specimen data). Alcohol or formalin field fixation was determined using associated field data and the physical condition of the voucher specimens, as well as testing residual formalin in liquid media with MQuant Formaldehyde Test Strips (Merck) to confirm presence of formaldehyde (Table S1). Thirty‐three of the specimens (nine alcohol‐fixed, 14 formalin‐fixed, and 10 dried skins) collected after 1980 also had cryogenically preserved tissues (flash frozen in the field using liquid nitrogen), allowing direct comparisons between DNA sourced from a museum specimen with samples taken for the explicit purpose of DNA preservation.

**TABLE 1:** Sample numbers from each voucher preservation type and broad taxonomic groups.

Voucher type	Group	Number of samples (old/young)
Frozen tissues	Birds	16 (0/16)
	Squamates	9 (0/9)
	Amphibians	8 (0/8)
Dried skins	Birds	42 (32/10)
	Squamates	NA
	Amphibians	NA
Skeletons	Birds	NA
	Squamates	5 (5/0)
	Amphibians	5 (5/0)
Alcohol‐fixed fluid specimens	Birds	6 (6/0)
	Squamates	11 (7/4)
	Amphibians	12 (6/6)
Formalin‐fixed fluid specimens	Birds	22 (10/12)
	Squamates	17 (10/7)
	Amphibians	12 (4/8)

*Note:* Sample numbers in brackets are the number of specimens grouped into young and old age groups. Detailed collection information about each sample is provided in Tables S1 and S2.

Each fluid specimen had a subsample of skin (toe pad for birds, abdominal skin for herpetological vouchers) and liver taken and subsamples of muscle and bone taken from the hindlimb or ribcage (in the case of legless specimens). Subsamples from fluid‐preserved specimens were then stored in 70% ethanol until extraction. For dry skins, which were solely represented by ornithological specimens, a toe pad was sampled, while for dried skeletons, a subsample of rib or phalange was collected.

### 2.2 DNA Extraction From Frozen Tissues

Frozen tissue samples were subsampled and genomic DNA was extracted using a Qiagen DNeasy Blood & Tissue kit following standard protocols, as described in Salis et al. (2025). Briefly, tissues were processed using manufacturer‐recommended digestion and purification steps, with DNA recovered via a double elution in AE buffer to maximize yield.

### 2.3 DNA Extraction From Voucher Specimens

All pre‐PCR steps (i.e., extraction, library preparation) for historical museum voucher specimens were conducted in purpose‐built historical DNA (hDNA) facilities at the American Museum of Natural History (AMNH), separate and physically isolated from any other molecular laboratories. A number of precautions were taken following standard protocols for the analysis of aDNA (e.g., decontamination of surfaces, reagents and materials, inclusion of negative controls) to minimize contamination of samples with exogenous DNA (Cooper and Poinar 2000; Knapp et al. 2012).

Tissue subsamples from all voucher specimens were weighed, minced, washed briefly with 70% ethanol, and then washed twice with ddH_2_O. Whole genomic DNA was extracted using a modified Qiagen DNeasy Blood & Tissue protocol with QIAquick spin columns to improve recovery of short DNA fragments. The same degraded‐DNA‐optimized extraction protocol was used for all voucher specimens, including formalin‐fixed material, to allow direct comparisons of DNA quantity and quality across voucher types and tissues rather than optimization for any one preservation class. Full extraction details are provided in the Supporting Information Methods.

### 2.4 DNA Quantity and Quality Assessment

To measure DNA yield, fluorescence was measured using a High Sensitivity Assay Qubit, Invitrogen, Waltham, MA, using 1–5 μL aliquots. Elution volumes differed among extraction protocols. DNA yield was therefore calculated as total DNA recovered (ng), accounting for elution volume, and normalized by tissue input mass (ng DNA per mg tissue). For frozen tissues, DNA fragment length distributions were analysed using Genomic DNA ScreenTapes on an Agilent 4150 TapeStation System. For historical specimens, DNA fragment length distributions were analysed using either D1000 or High Sensitivity D1000 ScreenTapes dependent on Qubit concentrations. For those younger historical specimens with fragment lengths out of range of the D1000 tapes, fragment analysis was rerun on a Genomic DNA ScreenTape. Together, Qubit‐derived DNA yield and TapeStation fragment‐size distributions represent extract‐level measures of total DNA, which may include both endogenous and exogenous DNA.

### 2.5 DNA Library Preparation and Sequencing

To minimize library preparation method biases, all modern and historical DNA extracts were prepared using the BEST protocol (Carøe et al. 2018) with modifications as per Mak et al. (2017). Modern extracts and historical extracts with large fragment sizes (> 1000 bp peak fragment size) were concentrated and sheared before library preparation. Library input DNA was not normalized by concentration; instead, a fixed volume of extract was used for all libraries, so absolute DNA input varied among samples depending on extract concentration. Libraries were indexed and amplified in duplicate PCRs, pooled, cleaned, quantified, and sequenced as 150 bp paired‐end reads on Illumina HiSeq X Ten or NovaSeq 6000 platforms. Full library preparation and indexing PCR details are provided in the Supporting Information Methods.

### 2.6 Data Processing

All sequencing data were processed using the EAGER pipeline v 2.5.1 (Fellows Yates et al. 2021) with NextFlow v 22.10.7 (Di Tommaso et al. 2017). Adapter sequences were removed and paired‐end reads collapsed using ADAPTER REMOVAL v2.3.2 (Schubert et al. 2016), trimming bases below a minimum Phred quality score of 10 and discarding reads shorter than 15 bp after clipping. Read quality was visualized before and after adapter trimming using fastQC v0.11.9 (http://www.bioinformatics.babraham.ac.uk/projects/fastqc/) to ensure efficient adapter removal. Reads were then mapped against a reference genome of the corresponding species or a reference of the closest related species (Table S1) using BWA v 0.7.17‐r1188 (aln ‐l 1024, ‐n 0.01, ‐o 2; Li and Durbin 2009). BAM files were then filtered using SAMTOOLS v1.12 (Li et al. 2009), filtering out unmapped reads and removing reads mapping with a mapping quality score (MAPQ) less than 15. PCR duplicates were removed using MARKDUPLICATES from the Picard package v 2.26.0 (http://broadinstitute.github.io/picard/). Damage profiles were generated using DamageProfiler v 0.4.9 (Neukamm et al. 2021), also calculating cytosine nucleotide misincorporation rates at the 5′ as an indication of DNA damage (cytosine deamination). Endogenous content was estimated using endorS.py (https://github.com/aidaanva/endorS.py) as the proportion of retained quality‐filtered reads mapping to the reference genome prior to PCR duplicate removal. Median fragment length distributions were additionally estimated from mapped sequencing reads by calculating the length of aligned read pairs following quality filtering.

As several specimens did not have a close representative nuclear reference genome (e.g., not within the same genus), reads were additionally mapped to mitochondrial reference genomes (Table S1), for which nearly every specimen had a representative genome from the same species or at least the same genus. Mitochondrial DNA mapping was performed using the same EAGER/BWA aln workflow, parameters and filtering as nuclear DNA mapping. Mitochondrial DNA endogenous content was calculated using the same denominator as nuclear DNA endogenous content: the proportion of retained quality‐filtered input reads mapping to the reference genome (i.e., the mtDNA genome). These mitochondrial DNA estimates were used as complementary sensitivity analyses because closer references were more consistently available. Hereafter, endogenous DNA refers to nuclear endogenous DNA unless stated otherwise.

### 2.7 Statistical Analysis

We tested the impact of various factors on: (1) DNA quantity and quality; (2) DNA degradation over time; and (3) DNA quality by tissue source. For DNA quantity and quality across all museum voucher types, we independently modelled five response variables (DNA yield per unit of mass of tissue, peak DNA fragment size, mapped median read length, estimated endogenous content, and the average percentage of cytosine deamination across the first two bases at the 5′ end of the read) using generalized linear models in the R statistical platform v 4.3.1 (R Core Team 2024). Pairwise post hoc comparisons were performed using estimated marginal means with Tukey adjustments of *p*‐values in the R package emmeans v 1.10.6 (Lenth 2024) to test for differences between different levels of each factor. To test for relationships in DNA degradation over time, the same five measures of DNA quality and quantity were modelled versus specimen age using linear regressions. Only samples from dry skins, formalin‐fixed, and alcohol‐fixed specimens were included in this analysis as skeletons and frozen tissues lacked sufficient sampling across the same time period. Post hoc pairwise comparisons were performed using R package emmeans v 1.10.6 (Lenth 2024) to test for differences in the trends between specimen age and the response variable (e.g., slope of linear regression) between different preservation types (Table S3).

Our primary statistical analyses were conducted using mapped read length, endogenous content, and the percentage of cytosine deamination at the 5′ end of the read estimates derived from nuclear genome mappings across all samples. To evaluate the robustness of these results to potential sources of reference bias, we performed three complementary analyses. First, analyses were repeated using a restricted dataset including only specimens with a close representative nuclear reference genome (a reference genome at least within the same genus), yielding qualitatively identical results (Figure S1). Second, to minimize reference genome divergence, reads were additionally mapped to mitochondrial reference genomes (for which all specimens had a reference genome within the same genus) and used to generate complementary estimates of mapped read length, mitochondrial endogenous content, and cytosine deamination (Figure S2). Third, to assess the effect of uneven sequencing effort among libraries, reads from high‐coverage libraries were randomly downsampled three times to different read depths, processed as above, and key metrics recalculated, showing high stability across sequencing depths (Figure S4).

For specimens that had both a voucher specimen and a frozen tissue (taken before fixation) paired sample *t*‐tests were run within R to directly compare DNA quantity and quality under ideal preservation conditions (frozen) and museum conditions (either dried‐skin, alcohol‐fixed, or formalin‐fixed) across the same individual. To deduce the tissue DNA source that performed the best for fluid‐preserved voucher specimens, alcohol and formalin‐fixed specimens were analysed separately using generalized linear models in R to test for differences in response variables with respect to tissue type, taxonomic group, and age group. To better evaluate the relative performance of tissue types within each specimen, response values for each tissue (e.g., DNA yield, fragment size, endogenous content) were divided by the specimen‐specific mean across all sampled tissues. For all models, preservation method, taxonomic group, and age group (young vs. old), and their potential interactions were included as explanatory variables. Post hoc comparisons were performed using the R package emmeans v 1.10.6 (Lenth 2024) to test for differences between tissue types within each taxonomic group and age group (old vs. young).

## 3 Results

Measurable DNA was extracted from all museum specimens irrespective of voucher type, ranging from 0.37–1564 ng of DNA per mg of tissue in alcohol‐fixed specimens, 0.29–580 ng/mg in formalin‐fixed specimens, 0.25–118 ng/mg in dried skeletons, 7.35–1160 ng/mg in dried skins, and 2.22–1125 ng/mg in frozen tissues. Fragment sizes varied from 45–5336 bp in alcohol‐fixed specimens, 45–937 bp in formalin‐fixed specimens, 73–192 bp in dried skeletons, 96–14,556 bp in dried skins, and 528–58,884 bp in frozen tissues. Endogenous content estimates from sequencing revealed that 13 of the alcohol‐fixed (eight old and five young) and 20 of the formalin‐fixed specimens (12 old and eight young) had endogenous content estimates less than 5% across all four sampled tissue types. Endogenous content of fluid‐preserved specimens varied widely, 0.1%–83.54% ($\bar{x}$ = 20.06%) in alcohol‐fixed specimens and 0.05%–89.91% ($\bar{x}$ = 18.39%) in formalin‐fixed specimens. Dry specimens and frozen tissues returned endogenous content estimates ranging from 2.58% to 82.66% ($\bar{x}$ = 44.32%) in skeletons, 11.24%–46.76% ($\bar{x}$= 29.03%) in skins, and 16.24%–85.89% ($\bar{x}$ = 65.21%) in frozen tissues. Endogenous content was primarily estimated from nuclear genome mappings; complementary analyses using mitochondrial mappings, restricted reference sets, and read downsampling are reported in the Supporting Information (Figures S1–S4).

Voucher type and taxonomic group had a significant impact on all response variables (Table 2: all *p*‐values < 0.01). Voucher type had a large effect size (all > 0.14) across all four response variables and had the largest effect size for DNA yield, fragment size, and cytosine deamination (Table 2). The effect sizes for voucher type and taxonomic group were both large for endogenous content, likely owing to biases in the estimation of endogenous content between taxonomic groups. For example, there are evolutionary closer reference genomes for bird specimens compared to those for amphibians and non‐avian reptiles (hereafter referred to as reptiles). For all other variables, taxonomic group only had a small effect size (Table 2).

**TABLE 2:** Statistical results of generalized linear models assessing DNA quantity and quality across voucher types, taxonomic groups, and collection year.

Explanatory variable	DNA yield	Peak fragment size	Endogenous content	C to T deamination 1st base
***Voucher type***	< 2.2e−16*** (0.35)	< 2.2e−16*** (0.80)	< 2.2e−16*** (0.39)	< 2.2e−16*** (0.32)
*Taxonomic group*	4.20e−3** (0.03)	6.741e−3** (0.03)	< 2.2e−16*** (0.28)	3.55e−3** (0.04)
*Collection year*	3.65e−11*** (0.11)	5.63e−4*** (0.03)	0.11 (6.67e−3)	< 2.2e−16*** (0.28)
*Voucher type: Taxonomic group*	0.06 (0.03)	1.07e−12*** (0.15)	2.10e−12*** (0.14)	9.14e−3** (0.05)
*Voucher type: Collection year*	4.81e−16*** (0.17)	7.84e−15*** (0.17)	6.25e−6*** (**0.07**)	0.23 (0.01)
*Collection year: Taxonomic group*	0.91 (4.91e−04)	0.87 (7.48e−4)	6.13e−6*** (0.06)	0.66 (2.14e−3)

*Note:* The table reports *p*‐values and partial eta squared effect sizes for each explanatory variable and their interactions across four DNA metrics: DNA yield, peak fragment size, endogenous content, and C to T deamination at the 5′ end. Statistical significance is indicated by bolding. *p*‐values < 0.05, 0.01, and 0.001 are indicated by *, **, and *** respectively. Effect sizes measured as partial eta squared are given in brackets for each measure in the model.

Collection year had a significant impact on DNA yield, fragment size, and cytosine deamination, with a large effect size for cytosine deamination (Table 2). No significant relationship between endogenous content and collection year was found. The interaction between voucher type and collection year had a significant impact on all response variables except cytosine deamination, with small to large effect sizes (Table 2). The interaction between voucher type and taxonomic group had a significant impact on fragment size, endogenous content, and cytosine deamination, both with large effect sizes on fragment size and endogenous content, while the interaction between collection year and taxonomic group was significant only for endogenous content with a moderate effect size (Table 2).

### 3.1 DNA Quantity and Quality Across Voucher Specimens

Frozen tissues preserved the highest quantities, longest fragment lengths, and highest proportions of endogenous DNA (Figure 1a). However, when separating voucher specimens into young and old groups (frozen only represented by young specimens, and skeletons only by old specimens), younger bird skins tended to preserve higher amounts of DNA than frozen tissues and all other voucher types; however, post hoc comparisons revealed the difference between young skins and frozen tissues was not significant (*p*‐value = 0.14). Despite preserving potentially higher DNA yields, younger skins preserved significantly smaller fragment lengths than frozen tissues (*p*‐value < 0.05). In all voucher specimens with young and old specimens, older specimens preserved less DNA, with smaller fragments. Surprisingly, in both types of fluid‐preserved specimens, no significant difference between the proportion of endogenous content between older and younger specimens was recovered. Notably, dry skins had higher DNA yields and larger fragment sizes even in old specimens when compared to fluid preserved specimens.

**FIGURE 1:**
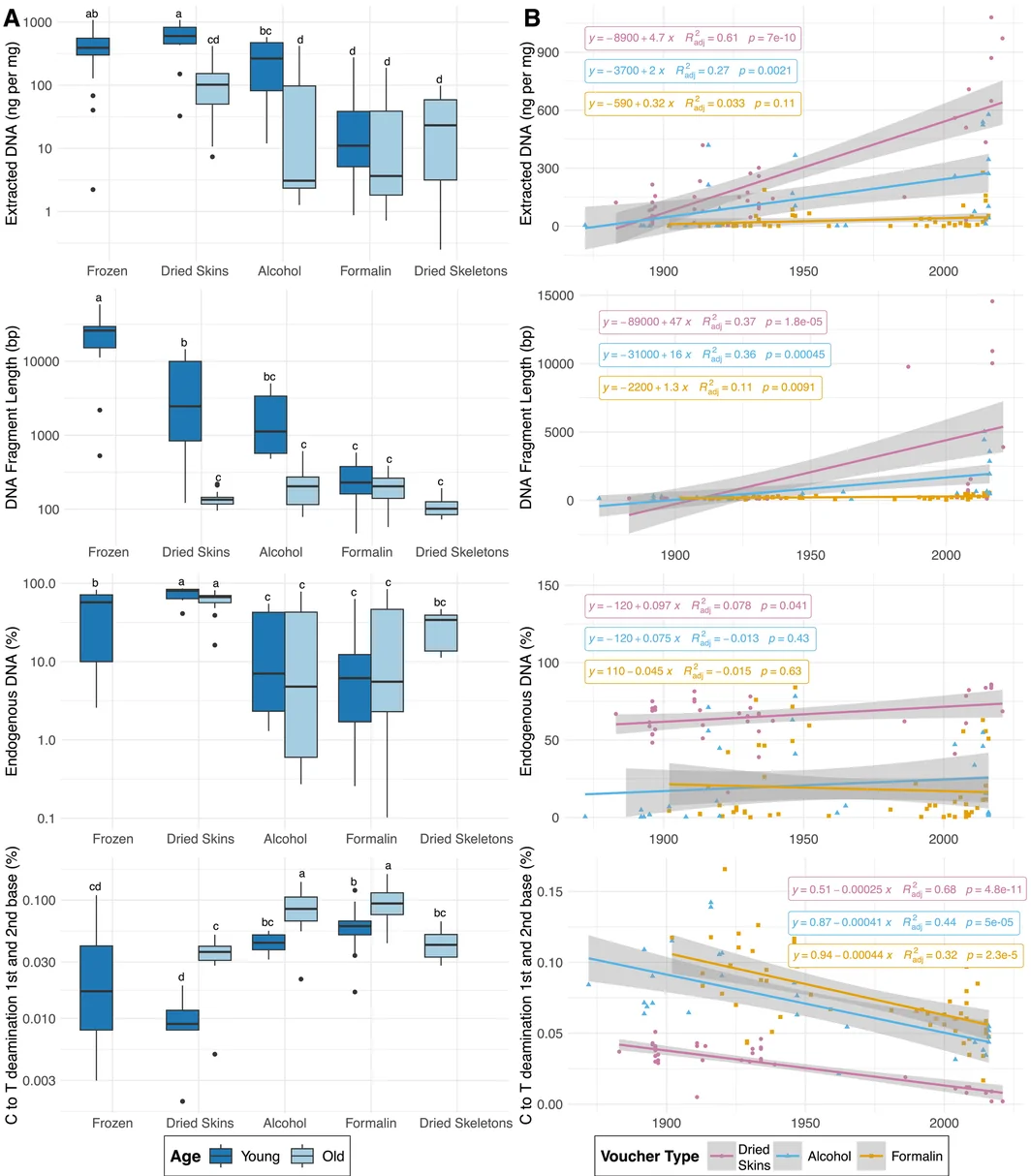
Effect of museum voucher preservation method on measures of DNA quality and quantity. DNA yield per unit mass of tissue, DNA peak fragment size, endogenous content; the fraction of sequenced reads mapping to the reference genome, and misincorporation of cytosine at the 5′ end of reads, with specimen age either: (A) treated as a categorical variable binned into age categories of young (1980–present) or old (1870–1980) or (B) age treated as a continuous variable in linear regression models. Letters above boxplots indicate post hoc statistical groupings; groups sharing a letter are not significantly different. Note that the *Y*‐axis is a log scale in (A) and linear scale in (B).

As a final measure of DNA damage and quality, cytosine to thymine (C to T) post‐mortem damage derived misincorporations at 5′ ends of sequenced reads (cytosine deamination) was explored (Figure 1a). These transitions are primarily caused by the deamination of cytosine to uracil (Briggs et al. 2007; Dabney et al. 2013), which is subsequently read as thymine during sequencing. The increased degradation of DNA in fluid‐preserved specimens was further reflected in an increase in cytosine misincorporations at 5′ ends of sequenced reads (Figure 1a). Further, frozen tissues and dried skins exhibited the lowest levels of cytosine misincorporation; older specimens exhibited higher misincorporation levels in all cases. While dried skeletons overall had the highest average misincorporation levels, it was significantly lower than in older fluid‐preserved specimens (Figure 1a).

Patterns inferred from fragment size estimated directly from extracts (TapeStation) were mirrored by sequencing‐derived fragment length estimates, with median mapped read lengths showing similar differences among preservation methods (Figure S3). Qualitative patterns of endogenous content across preservation methods were largely unchanged when analyses were restricted to specimens with close nuclear reference genomes (Figure S1), and when endogenous content was estimated from mitochondrial mappings (Figure S2), despite differences in absolute values. Mitochondrial DNA mapping also showed an increase in endogenous content with sample age in dried skins (Figure S2). Downsampling analyses of high‐coverage libraries showed that endogenous content, mapped read length, and cytosine deamination estimates were stable across a wide range of sequencing depths (Figure S4).

### 3.2 Measures of DNA Over Time

Comparing DNA quantity and quality over time revealed differences in relation between DNA preservation and time between dry skin specimens and fluid‐preserved specimens (Figure 1b). In dried skins there was a clear reduction in DNA yield and fragment size with increasing age; younger skins (younger than 30 years) routinely recover fragment lengths greater than 1000 bp, where older specimens are highly fragmented with fragment lengths often below 300 bp (Figure 1). In alcohol‐fixed specimens there was a clear trend of decreasing DNA yield and fragment length over time (DNA yield: *R* ^2^ = 0.27, *p*‐value = 0.0021; fragment length: *R* ^2^ = 0.36, *p*‐value = 4.5e−4; Figure 1b), however, the slope of the regression was significantly reduced compared to dried skins (DNA yield: *p*‐value = 0.0003; fragment length: *p*‐value = 0.0017; Table S3), indicating a breakdown in the association over time. This decoupling between the natural fragmentation of DNA and degradation of DNA over time was further exaggerated in formalin‐fixed specimens (Figure 1b; Table S3), and remarkably, there was no significant difference in DNA yield and fragment lengths between younger and older specimens (Figure 1a), with linear regressions proving a poor fit (DNA yield: *R* ^2^ = 0.04; fragment length: *R* ^2^ = 0.11). This pattern was consistent when fragment size was assessed using median mapped read length (Figure S3), indicating that the lack of time‐dependent fragmentation in formalin‐fixed specimens was not an artefact of extract‐based size distributions alone.

Although less exaggerated, there was a decrease in endogenous DNA over time in dry skin specimens (*R* ^2^ = 0.078, *p*‐value = 0.041); however, in the fluid‐preserved specimens, neither showed a significant correlation over time (alcohol‐fixed *p*‐value = 0.43, formalin‐fixed *p*‐value = 0.63). Alcohol‐fixed specimens may show a weak negative relationship with age, but in formalin‐fixed specimens this association was completely absent (Figure 1b). Conversely to a decrease in the relationship with time in other response variables, cytosine misincorporations showed an increased relationship with time, especially in fluid‐preserved specimens (Figure 1b). The relationship between cytosine misincorporations and time appeared to be strong in fluid‐preserved specimens (alcohol‐fixed: *R* ^2^ = 0.44, *p*‐value = 5e−5; formalin‐fixed: *R* ^2^ = 0.32, *p*‐value = 2.3e−5). While in dried skins, there was still a strong association with time (*R* ^2^ = 0.68, *p*‐value = 4.8e−11), the slope appeared reduced compared to fluid‐preserved specimens, although not significant (skin‐alcohol‐fixed: *p*‐value = 0.24; skin‐formalin‐fixed: *p*‐value = 0.23; Table S3), with less difference in the cytosine misincorporation between younger and older specimens.

Linear regression analysis showed that collection date explained a significant proportion of variation in DNA yield, fragment length, and cytosine deamination (Figure 1b). Notably, collection date explained more of the variation in dry skin specimens than in alcohol‐ or formalin‐fixed specimens across DNA yield (*R* ^2^ = 0.61 vs. 0.27 and 0.033 for alcohol‐ and formalin‐fixed, respectively) and fragment length (*R* ^2^ = 0.37 vs. 0.36 and 0.11). Linear models for endogenous content were, in general, poorly fit (Figure 1b), with only a very weak association of endogenous content with collection date in dry skin specimens (*R* ^2^ = 0.078).

### 3.3 Paired Comparison Between Frozen and Voucher Specimens

Unsurprisingly, for nearly every single measure, voucher specimens performed worse than frozen tissues, with smaller DNA fragments, lowered endogenous content (only significantly different in formalin‐fixed specimens), and increased C to T transitions at the end of reads due to cytosine deamination (Figure 2). Notably, in dried skins, the toepads tended to preserve higher yields of DNA than their frozen counterparts, although not significant.

**FIGURE 2:**
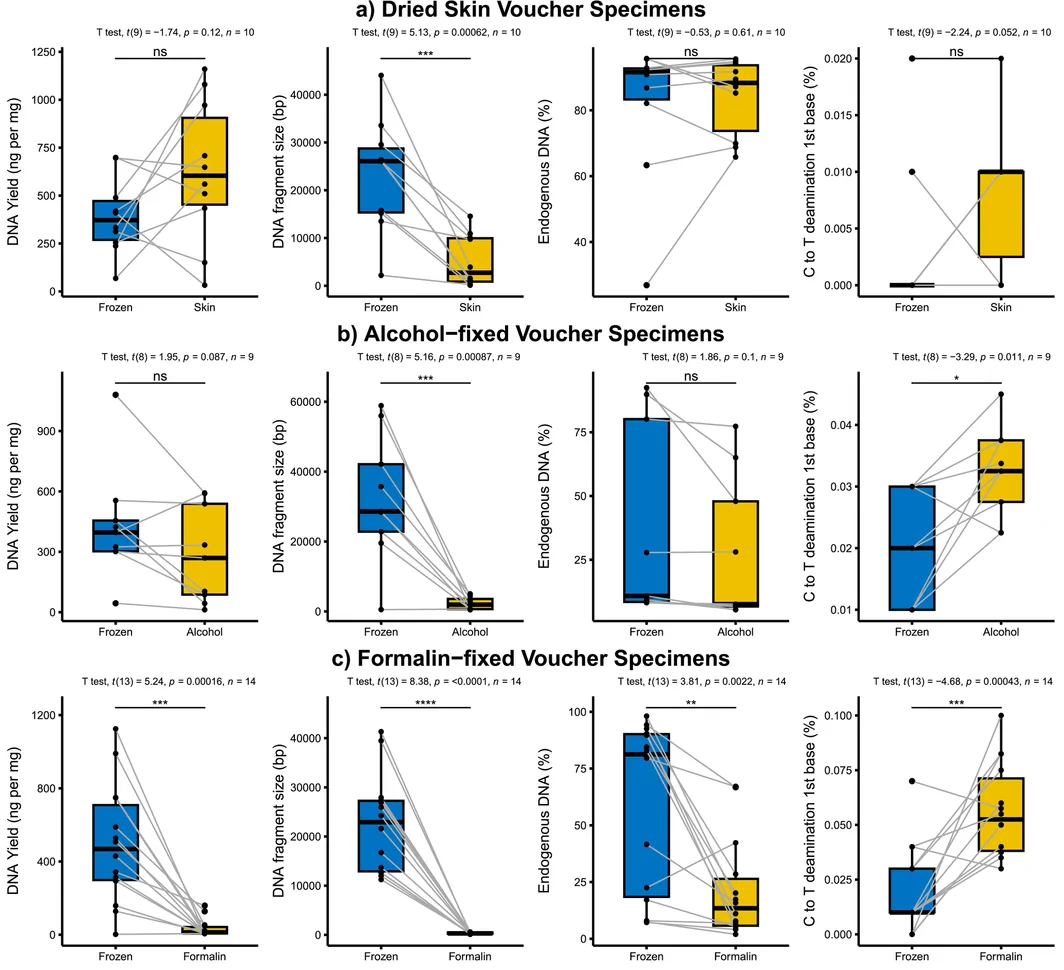
Paired comparisons of frozen tissue and different voucher type taken from the same individual: (a) dried skin vouchers, (b) alcohol‐fixed fluid‐preserved specimens, and (c) formalin‐fixed fluid‐preserved specimens, across four measures of DNA quality and quantity: DNA yield per unit mass of tissue, DNA peak fragment size, endogenous content—the fraction of sequenced reads mapping to the reference genome, and misincorporation of cytosine at the 5′ end of reads due to deamination. Significance levels of t‐tests are as follows: ns *p* > 0.05, *p < 0.05, **p < 0.01, ***p < 0.001, ****p < 0.0001.

### 3.4 Tissue Performance in Fluid‐Preserved Specimens

When comparing the performance of different tissues in fluid‐preserved specimens, no single tissue source outperformed all others: results were highly dependent on taxonomic group, fixative, and age. Generalized linear models showed that tissue type and its interactions with age and taxonomic group had significantly impacted DNA yield in both alcohol and formalin‐fixed specimens, with large effect sizes (Table 3). Fragment size was less strongly affected by any of the explanatory variables, with tissue type significant only in alcohol‐fixed specimens. Tissue type significantly affected endogenous content in formalin‐fixed specimens, while in alcohol‐fixed specimens endogenous content and cytosine deamination were influenced only through interactions between tissue type, age group, and taxonomic group (Table 3).

**TABLE 3:** Results of generalized linear models evaluating tissue‐specific effects on DNA quality in alcohol‐ and formalin‐fixed specimens.

Explanatory variable	DNA yield	Peak fragment size	Endogenous content	C to T deamination 1st base
***Alcohol‐fixed specimens***				
***Tissue type***	7.47e−3** (0.11)	0.02* (0.09)	0.86 (7.65e−03)	0.54 (0.02)
*Taxonomic group*	1.00 (1.84e−21)	1.00 (2.11e−5)	1.00 (3.76e−21)	1.00 (6.97e−20)
*Age group*	1.00 (2.79e−22)	0.97 (9.40e−6)	1.00 (2.20e−21)	1.00 (2.16e−22)
*Tissue type: Taxonomic group*	4.64e−3** (0.16)	0.79 (0.03)	0.60 (0.05)	0.65 (0.04)
*Tissue type: Age group*	7.14e−4*** (0.15)	0.07 (0.07)	0.03* (0.08)	0.65 (0.02)
*Age group: Taxonomic group*	1.00 (2.20e−22)	1.00 (1.17e−7)	1.00 (1.60e−22)	1.00 (3.96e−23)
*Tissue type: Age group: Taxonomic group*	1.46e−3** (0.13)	0.32 (0.04)	0.12 (0.06)	0.26 (0.04)
***Formalin‐fixed specimens***				
***Tissue type***	< 2.2e−16*** (0.37)	0.14 (0.03)	2.32e−3** (0.08)	0.25 (0.02)
*Taxonomic group*	0.99 (1.31e−04)	0.94 (7.82e−4)	1.00 (6.96e−06)	1.00 (2.08e−6)
*Age group*	0.81 (3.23e−04)	0.92 (6.91e−05)	0.95 (2.35e−05)	0.97 (5.67e−06)
*Tissue type: Taxonomic group*	4.02e−06*** (0.17)	0.53 (0.03)	0.01** (0.09)	0.71 (0.02)
*Tissue type: Age group*	2.69e−08*** (0.18)	0.49 (0.02)	0.01* (0.06)	0.12 (0.02)
*Age group: Taxonomic group*	0.98 (2.51e−04)	0.95 (6.29e−4)	1.00 (1.15e−05)	1.00 (7.76e−06)
*Tissue type: Age group: Taxonomic group*	0.03 (0.07)	0.69 (0.02)	0.58 (0.03)	0.44 (0.03)

*Note:* Each preservation group was analysed separately for DNA yield, fragment size, endogenous content, and C to T deamination, including interactions with age group and taxonomic group. Statistical significance is indicated by bolding. *p*‐values < 0.05, 0.01, and 0.001 are indicated by *, **, and *** respectively. Effect sizes (partial eta squared) are given in brackets for each measure in the model.

In alcohol‐fixed specimens, liver preserved the highest quantity of DNA in younger specimens, whereas no single tissue outperformed the others in older specimens; bone tended to preserve more DNA, but this was not significant (Figure 3a). The pattern of liver preserving more DNA in alcohol‐fixed specimens was reflected in both amphibian and reptile specimens (there were no young alcohol‐fixed bird specimens). In older specimens, the tissue producing the highest DNA yield was largely dependent on taxonomic group, but with no significant differences, with liver and bone preserving more DNA in amphibians, skin and bone in birds, and muscle in reptiles (Figure S5a). Muscle tended to preserve larger DNA fragments overall in both younger and older specimens (Figure 3b). Within amphibians, bone, skin, and muscle appeared to preserve larger fragments in young specimens, while only muscle significantly preserved larger DNA fragments in older specimens (Figure S5b). In birds, muscle tended to preserve longer fragment lengths. In reptiles, no clear trends were seen, although skin in younger specimens tended to preserve larger fragments and bone smaller fragments (Figure S5b). In terms of endogenous content, no significant trend was found. However, skin appeared to preserve higher amounts of endogenous DNA in both young and old specimens, with liver also appearing to preserve higher amounts of endogenous DNA in older specimens (Figure 3c). Taxonomically, liver in amphibians preserved higher amounts of endogenous DNA in older specimens (up to 3 times as much), and muscle samples from reptiles tended to preserve lower amounts of endogenous DNA in older specimens, while in bird specimens, endogenous content was consistent across tissue types (Figure S5c). There were no clear trends or significant differences in cytosine deamination levels across alcohol‐fixed tissues, with bone appearing to have elevated cytosine deamination levels in young amphibian specimens, while muscle and liver showed higher levels of deamination in older amphibian specimens and muscle elevated levels in older reptile specimens (Figures 3d and S5d).

**FIGURE 3:**
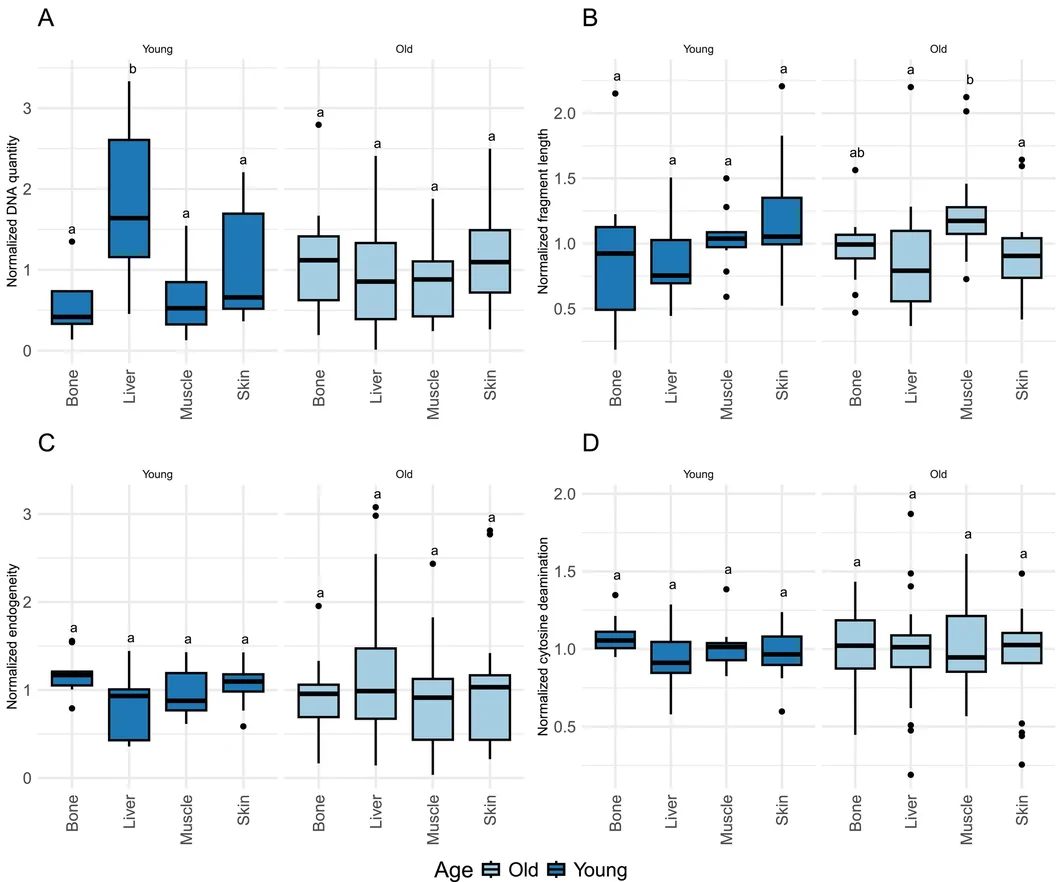
Effect of tissue type on measures of DNA quality and quantity in alcohol‐fixed specimens: (A) DNA yield per unit mass of tissue, (B) DNA peak fragment size, (C) endogenous content, the fraction of sequenced reads mapping to the reference genome, and (D) misincorporation of cytosine at the 5′ end of reads due to deamination. Specimens are further split by age group: Young (1980–present) or old (1870–1980). Results of post hoc Tukey tests within each level of age are represented by letters above the boxes. For results split by taxonomic group see Figure S5.

Formalin‐fixed specimens showed strikingly different patterns compared to alcohol‐fixed specimens (Figure 4). Higher DNA yields were obtained from liver in younger specimens, while more DNA was obtained from skin in older specimens (Figure 4a). This pattern was observed in both amphibians and birds; however, in reptiles, more DNA was obtained from liver in both younger and older specimens (Figure S7a). Overall, there were no overwhelming trends in fragment length patterns, although smaller fragments tended to be obtained from liver in older specimens (Figure 4b). This trend was especially true in amphibian and bird specimens (Figure S7b). Further, in younger specimens of amphibians, smaller fragments were obtained from liver and higher fragment lengths in muscle. In older bird specimens, higher fragment lengths tended to be obtained from skin (Figure S7b). In terms of endogenous content, higher proportions of endogenous content tended to be obtained from skin samples, although not significant (Figure 4c). In younger specimens, more endogenous content tended to be obtained from bone and skin, with less obtained from muscle and liver (Figure 4c). The higher content in younger bone seems to be largely driven by bird and reptile specimens and was absent in amphibians, while the lowered endogenous content of muscle was consistent across all groups (Figure S7c). Amphibian and reptile specimens further showed lower endogenous content in older bone samples. There were no clear trends or significant differences in cytosine deamination levels across formalin‐fixed tissues, with the exception that skin in older specimens tended to have lowered levels of cytosine deamination, and in amphibian specimens, bone exhibited the highest and liver the lowest deamination levels in young specimens (Figures 4d and S7d).

**FIGURE 4:**
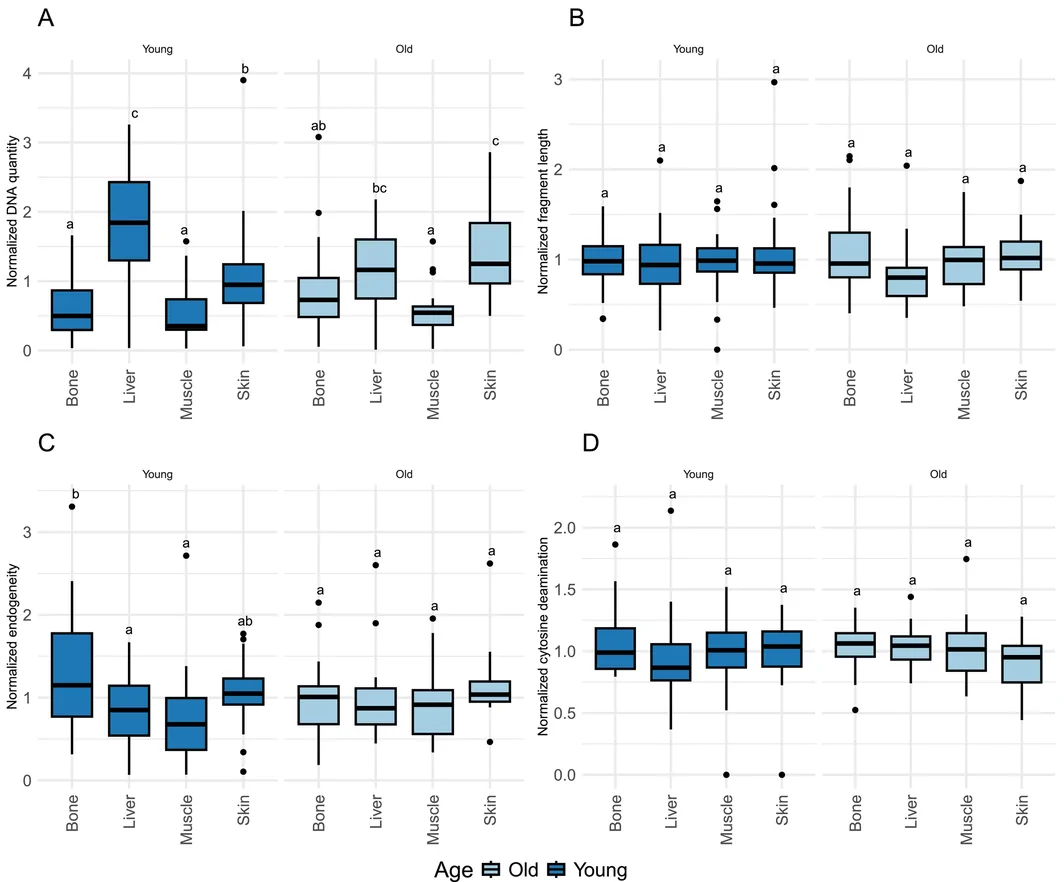
Effect of tissue type on measures of DNA quality and quantity in formalin‐fixed specimens: (A) DNA yield per unit mass of tissue, (B) DNA peak fragment size, (C) endogenous content, the fraction of sequenced reads mapping to the reference genome, and (D) misincorporation of cytosine at the 5′ end of reads due to deamination. Specimens are further split by age group: Young (1980‐present) or old (1870–1980). Results of post hoc Tukey tests within each level of age are represented by letters above the boxes. For results split by taxonomic group see Figure S7.

## 4 Discussion

Our comparative framework showed that degradation profiles of fluid‐preserved specimens differed from other specimen types. In fluid‐preserved specimens, we observed the expected pattern of smaller fragments, decreased endogenous DNA, and increased deamination with age in some tissue types, but declines in DNA yield or fragment length over time in formalin‐fixed specimens were absent. These patterns show formalin fixation both rapidly degrades and stabilizes DNA whereas in dry specimens DNA degradation more generally increases with time. Across tissue types in fluid‐preserved specimens, our results showed that the optimal tissue type depended on specimen age and fixation history. While there was substantial variation among specimens and tissue types, our findings can be used to inform decision‐making in identifying the optimal sources of DNA for museomic research. Because DNA yield and TapeStation fragment‐size distributions are extract‐level metrics that may include both endogenous and exogenous DNA, we interpreted them alongside sequencing‐derived measures of mapped read length, endogenous content, and cytosine deamination rather than as standalone indicators of genomic utility.

### 4.1 DNA Degradation Profiles

Our study demonstrates that fluid‐preserved specimens—particularly those preserved in alcohol and even some older formalin‐fixed specimens—can yield substantial quantities of DNA, with a subset producing high endogenous content (> 70%) and large enough fragments for genome‐wide analyses. Importantly, we identify liver as the best DNA source in younger specimens and skin and muscle as more reliable tissues in older specimens, depending on taxonomic group and fixative. However, we also note that there is substantial variation between individual specimens of the same preparation type and age, indicating that the collection and archive history of specimens may also be having a major influence on DNA degradation.

Building from this, we benchmarked the performance of fluid‐preserved specimens relative to other commonly used museum vouchers. As expected, frozen tissues produced the highest DNA yields, largest fragment lengths, and preserved the highest proportion of endogenous DNA. This was followed by dried skin voucher specimens, which also preserved higher DNA yields, larger fragment lengths, and higher proportions of endogenous content than all other voucher type specimens not specifically prepared to preserve biomolecules. Further, paired comparisons between frozen tissues and voucher specimens prepared from the same individual before fixation showed that traditional vouchers generally show lower yields of highly fragmented DNA with lower endogenous proportion (i.e., an increase in exogenous contamination) and an increase of C to T transitions due to cytosine deamination.

Surprisingly, toe pads from younger dried skins tended to have higher DNA yields and endogenous content than even frozen tissues. Some of this pattern is likely due to taxonomic effects of frozen tissues being represented by all three taxonomic groups while skins were solely represented by bird specimens which typically preserved higher DNA yields and have reference genomes from more closely related taxa. Many amphibian and reptile specimens did not have available references within the same genus let alone species, potentially biasing the performance results of skins. Where paired comparisons were available between dried skins and frozen tissues, there was no significant difference in endogenous content, supporting the hypothesis that this observation was likely due to taxonomic biases. Toe pads are heavily keratinized tissues and are known to be a reliable source of historical DNA in birds and mammals (Irestedt et al. 2022; Roycroft et al. 2022; Tsai et al. 2020), with keratin‐rich tissues potentially being protected from DNA damage (Bengtsson et al. 2012; Topfer et al. 2011). Our frozen tissues were represented by muscle, which may naturally contain less DNA per unit mass than toe pads and therefore over the shorter time period represented by these tissue pairs, toe pads from dried skins are still able to achieve high yields, although with significant signals of degradation (highly fragmented). Despite this issue, the results clearly show dried skin voucher specimens are highly amenable to museomic research (McDonough et al. 2018; Mundy et al. 1997; Pacheco et al. 2022; Tsai et al. 2020).

Because endogenous content and damage estimates can be influenced by the evolutionary distance of the reference genome used for mapping, we evaluated whether the observed performance of voucher types was robust to reference choice. When analyses were restricted to specimens with close nuclear reference genomes (reference genome within the same genus), dried skins continued to outperform other voucher types, exhibiting higher endogenous content and lower damage levels, indicating that these patterns are not driven completely by uneven reference availability. Similarly, when reads were mapped to mitochondrial genomes, no significant differences in mitochondrial endogenous content were detected among voucher types; however, reads derived from dried skin specimens consistently exhibited lower levels of cytosine deamination. Notably, mitochondrial DNA endogenous content increased with specimen age in dried skins, a pattern not mirrored in nuclear DNA estimates. Because mitochondrial DNA endogenous content was calculated as a proportion of all retained reads, this pattern may be partly compositional: recent dried skins with higher nuclear DNA endogenous content may show lower mitochondrial DNA mapping proportions even if mitochondrial DNA preservation is not poorer. Similar compartment‐specific patterns have also been reported in ancient and historical material, where nuclear DNA loss may proceed more rapidly than mitochondrial DNA loss over time, potentially reflecting structural protection of mitochondrial genomes (Allentoft et al. 2012; Higgins et al. 2015; Schwarz et al. 2009). Although mitochondrial genome references were generally closer than available nuclear genome references, interspecific divergence within genera may still reduce mapping efficiency and preferentially recover more conserved regions of the mitochondrial genome. As a result, mitochondrial DNA mapping metrics should be interpreted cautiously and not assumed to directly reflect nuclear genomic recovery in degraded samples.

Dried skeletons, on average produced poor results, although this was clearly biased by this voucher type being solely represented by older specimens as unfortunately no younger specimens were used in this study. When compared solely to other older specimens, dried skeletons generally outperformed fluid specimens and were comparable in performance to dried skins, with a higher proportion of endogenous content, but slightly lower DNA yield. Despite being only represented by older specimens, our results recapitulate previous studies that find skeletons are a reliable source of DNA for museomic research (McDonough et al. 2018; Pacheco et al. 2022; Stuart et al. 2006). Nevertheless, sampling of skeletons is generally viewed as much more destructive than sampling of other voucher types (e.g., skins), meaning sampling will only be beneficial if alternative sample types are not available.

When accounting for sampling bias in the skeleton dataset, fluid‐preserved specimens performed the worst of all museum voucher specimens, with lower DNA yields, smaller DNA fragments, lower proportion of endogenous DNA, and increased cytosine deamination at the end of reads. Yet it is an intriguing finding that older fluid‐preserved specimens tended to preserve larger DNA fragments than dried skins or skeletons of the same age. This could indicate that, over longer periods of time, fluid‐fixation may protect against DNA fragmentation—but it is more likely these specimens had a higher proportion of high molecular weight DNA contamination, as evidenced by their lower estimates of endogenous DNA content.

In our study, despite their overall lower performance, fluid‐preserved specimens yielded > 70% endogenous content in 21 cases, including some formalin‐fixed samples from the 1950s or earlier. Most notably, five specimens were all fixed in formalin and predate the 1950s but retrieved endogenous content estimates > 70% in at least one tissue. These samples would be highly amenable to more in‐depth museomic studies and are excellent candidates for deeper sequencing and whole genomic analysis. While most of the specimens returned lower endogenous content estimates (< 20%), these specimens would still be viable for museomic research, especially when using capture‐based approaches (e.g., Bernstein and Ruane 2022; O'Connell et al. 2022; Ruane and Austin 2017; Wood et al. 2018). Using < 1% endogenous content as a conservative heuristic for specimens likely to be challenging for capture‐based approaches, approximately 18% (14/80) of fluid‐preserved specimens in this study would be flagged, although specimens below this value may still be successful depending on sequencing depth, fragment length, and target‐capture strategy. For target‐capture approaches such as ultraconserved elements (UCEs), samples dominated by short fragments may recover conserved core regions while losing more informative flanking sequence, meaning that capture success may not always translate into phylogenetically useful data (Abreu et al. 2022; Faircloth et al. 2012; Hinckley, Pinilla‐Buitrago, et al. 2025; Hosner et al. 2016). However, it is important to note that this estimate is likely to be an overestimate as 50% of the failed specimens were microhylid specimens, where the closest available reference ( *Gastrophryne carolinensis* ) is approximately 45–55 mya diverged (Portik et al. 2023), meaning the endogenous content estimates of such specimens is likely to be highly biased by a lack of suitable reference genomes. Thus, fluid‐preserved specimens—though highly variable—offer a more reliable source of viable DNA than currently appreciated, particularly for understudied taxa.

### 4.2 Time‐Dependent Patterns

The recovery of high molecular weight DNA (> 1000 bp) from some younger voucher specimens—including those preserved as dried skins or fluid‐preserved samples—suggests that selected archival specimens may also be suitable for emerging long‐read sequencing approaches. However, these approaches have historically relied on fresh and frozen tissues, limiting their use in taxa that are difficult to sample or already extinct (Blom 2021). Despite the difficulties associated with DNA quality, long‐read sequencing has been successfully applied to some natural history collections, including ethanol preserved blood samples (Bein et al. 2025) and herbaria specimens (Quatela et al. 2023). With appropriate pre‐screening, library preparation, and sequencing strategies, selected voucher specimens could therefore help close phylogenetic gaps and expand high‐quality genomic representation across the tree of life.

One of the most striking results was the absence of expected time‐dependent DNA fragmentation in fluid‐preserved specimens, especially those fixed with formalin. Fragment length in formalin‐fixed specimens showed no significant association with age, suggesting that much of the observed DNA fragmentation occurs early during fixation. This is consistent with FFPE studies showing substantial DNA fragmentation during the initial stages of formalin exposure (Do and Dobrovic 2015; Hedegaard et al. 2014; Oh et al. 2015). The high level of fragmentation in younger formalin‐fixed specimens may contribute to the difficulty of extracting DNA from older formalin‐fixed specimens: further time‐dependent fragmentation could push molecules below the size range efficiently recovered by conventional extraction methods or confidently mapped to reference genomes (~25 bp). Under this scenario, a corresponding decline in DNA yield or endogenous content would be expected in older specimens, but this was not observed. The absence of a time‐dependent decline in fragment length for formalin‐fixed specimens was also observed when fragment size was estimated from mapped sequencing reads, indicating that this pattern is not driven solely by extract‐based size distributions or contamination. This pattern may reflect both rapid initial fragmentation and the potential for formalin‐induced crosslinks to stabilize nucleic acids against further enzymatic or hydrolytic degradation over time (Guyard et al. 2017; Srinivasan et al. 2002; Watanabe et al. 2017).

Despite the apparent breakdown of time‐dependent trends in DNA yield, fragment length, and endogenous content in formalin‐fixed specimens, cytosine deamination retained a clear positive relationship with specimen age. This likely reflects the continued accumulation of hydrolytic damage over time, as formalin fixation and long‐term storage in aqueous media maintain conditions that promote cytosine deamination even when other measures of DNA degradation are decoupled from specimen age (Lindahl 1993; Dabney et al. 2013; Do and Dobrovic 2015).

Although liver has been considered the superior source of DNA in fluid‐preserved specimens (Hahn et al. 2022; Hykin et al. 2015; Zacho et al. 2021), our tissue‐specific analyses show a more complicated story relating to both age, fixation method, and taxonomic group. Liver consistently yielded higher quantities of DNA in younger alcohol and formalin‐fixed specimens but did not clearly outperform other tissues in fragment length or endogenous content. In older specimens, liver performance declined, with skin, muscle, and to some extent bone performing better across several metrics. Instead, the best tissue is dependent on both taxonomic group and fixation methods, with skin as a clear out‐performer in older bird specimens. The only metrics where liver was a clear outperformer in older specimens were for DNA yield in formalin‐fixed reptiles and endogenous DNA proportion in alcohol‐fixed amphibians. Skin, muscle, and to some extent bone across all metrics performed better on average than liver in older specimens.

The poorer performance of liver in older fluid‐preserved specimens is consistent with the rapid postmortem degradation observed in freshly collected liver tissue (Amarilla‐Stevens et al. 2022), likely owing to high enzymatic activity and autolysis (Bar et al. 1988; Hanner et al. 2005). In contrast, muscle yielded lower quantities of DNA but often preserved larger DNA fragments. Because muscle did not tend to have lower endogenous content estimates, these longer fragments are unlikely to reflect high‐molecular‐weight contamination alone. It has been observed that muscle samples tend to be more resistant to DNA degradation than other tissue types in archival tissue collections (Amarilla‐Stevens et al. 2022; Camacho‐Sanchez et al. 2013). These results underpin the need to consider age and preservation method when sampling fluid‐preserved specimens, while although liver performs well for younger specimens, skin and muscle clearly outperformed liver on several metrics in older specimens. The use of liver may further explain the difficulty in retrieving reliable historical DNA data from older formalin‐fixed specimens (Appleyard et al. 2021; Brino et al. 2023; Hykin et al. 2015; Ruane and Austin 2017; Zacho et al. 2021), where sampling other tissues such as skin or muscle could increase chances of success.

All samples in this study were processed using a single extraction protocol to establish a consistent comparative baseline across tissues and preservation types. While this uniformity is a strength for cross‐category comparisons, it also means that some tissues—such as bone or keratinized skin—may not have been processed under conditions optimized for maximal DNA recovery. Similarly, because all voucher specimens were prepared using a double‐stranded library workflow, our results may provide conservative estimates of what can be recovered from highly fragmented or chemically modified material. Specialized extraction approaches, single‐stranded library preparation, damage‐tolerant workflows, or other low‐input methods may therefore improve recovery from challenging specimen classes, including formalin‐fixed tissues, consistent with work in invertebrate and vertebrate museomics showing that workflow optimization can be critical for historical DNA recovery (Derkarabetian et al. 2019; Hinckley, Flores, et al. 2025; Settlecowski et al. 2023).

Undocumented collection, preparation, and storage histories likely explain some variation among specimens of similar age and preservation type. Collector information was compiled where available, but was incomplete, sparse, and strongly confounded with age, voucher type, tissue type, and taxonomic group, precluding its inclusion as a formal model term. Factors such as delay before fixation, duration and anatomical distribution of formalin exposure, ethanol maintenance, field preparation practices, pest treatment, handling history, storage conditions, and tissue input mass may therefore affect DNA recovery in ways not fully captured by specimen age or voucher category. Because these factors vary among collections and institutions, the exact magnitude of degradation patterns may differ across museum systems, although the broad voucher and tissue‐level comparisons reported here provide a useful framework for anticipating DNA preservation in vertebrate collections.

### 4.3 Sampling Recommendations

Together, our results argue for a reevaluation of sampling priorities in fluid‐preserved specimens in museomic studies. Skin and muscle—being less invasive to sample than bone—emerge as promising alternatives to liver, especially in older specimens or when the preservation history is uncertain. Sampling both skin and underlying muscle (in a single sliver), especially from previously dissected or eviscerated specimens, may offer a practical way to improve historical DNA recovery. These findings challenge the assumption that liver represents the best chance of retrieval of historical DNA (Hahn et al. 2022; Hykin et al. 2015; O'Connell et al. 2022; Zacho et al. 2021), and demonstrate that even challenging fluid‐preserved specimens can be viable for genetic research when sampling is approached strategically. Our results also emphasize the general importance of specimen age for historical DNA recovery, with younger specimens producing higher quality DNA across most metrics with the exception of formalin‐fixed specimens and DNA fragment length.

Because many historical libraries are dominated by short inserts and variable endogenous content, pilot sequencing remains important for estimating whether a specimen is best suited to shallow screening, mitogenome assembly, target capture, or deeper shotgun sequencing. For libraries dominated by fragments < 100 bp, shorter paired‐end sequencing configurations (e.g., PE 75 bp) may also be more cost‐effective than longer read configurations.

## 5 Conclusion

By identifying the most reliable tissues for DNA extraction under various preservation methods and ages, we provide a framework for optimizing sampling strategies that balance maximizing extraction success with specimen conservation. Frozen tissues remain the optimal source for DNA preservation, but dried skins, skeletons, and fluid‐preserved specimens can all yield useful genetic data and expand the temporal and taxonomic reach of museomic research. Importantly, fluid‐preserved specimens, including formalin‐fixed material, should not be treated as uniformly poor DNA sources. Although they are variable and often degraded, many retained sufficient endogenous DNA for downstream genomic applications.

Our results also show that tissue choice matters. Liver may be an appropriate target in younger fluid‐preserved specimens, but its performance declines in older material, where skin and muscle often provide better alternatives. Given the substantial variation among specimens of similar age and preservation type, initial screening, duplicate sampling where possible, and improved documentation of field and collection histories will remain important for maximizing recovery while minimizing destructive sampling. Together, these findings reinforce the value of traditional voucher specimens, including fluid‐preserved material, as underused but viable resources for museomic research.

## Author Contributions

A.T.S., B.T.S., and C.J.R. designed research; A.T.S., N.A., S.K., and J.K. performed research; B.T.S. and C.J.R., contributed new reagents/analytic tools; A.T.S., N.A., S.K., J.K., B.T.S., and C.J.R. analysed data; A.T.S., B.T.S., and C.J.R. wrote the paper with input from N.A., S.K., and J.K.

## Funding

This research was funded by the U.S. National Science Foundation (DBI 2029955) awarded to C.J.R. and B.T.S.; and the Madagascar fieldwork supported by the U.S. National Science Foundation (DEB 1257610, 0641023, 0423286, 9984496, 9625873, 9322600 and BSR 9024505) to C.J.R.

## Disclosure

Benefit‐Sharing Statement: This research was in collaboration with scientists at different levels, from senior scientists to early career scientists (high school students), the latter of which received extensive training in molecular techniques and bioinformatics as a part of the project. The study tested common museum voucher specimens for DNA quality, which will help guide future genetic studies aiming to integrate museum specimens. All data are freely accessible as described above.

## Ethics Statement

No live animals were collected, handled, or euthanized for this study. All samples were obtained from existing museum specimens and associated frozen tissues held in the American Museum of Natural History Vertebrate Zoology collections under institutional curatorial approval. Therefore, no additional animal ethics approval was required.

## Conflicts of Interest

The authors declare no conflicts of interest.

## Supporting information


**Figure S1:** Effect of museum voucher preservation method on endogenous content and cytosine deamination when analyses are restricted to specimens with close nuclear reference genomes. Endogenous content (fraction of sequenced reads mapping to the nuclear reference genome) and cytosine to thymine misincorporation at the 5′ end of reads are shown with specimen age treated as either: (A) a categorical variable binned into young (1980–present) and old (1870–1965) age classes, or (B) as a continuous variable in linear regression models. Restricting analyses to specimens with close reference genomes does not qualitatively alter patterns among voucher types observed in the full dataset.
**Figure S2:** Endogenous content and cytosine deamination estimated from reads mapped to mitochondrial reference genomes. The proportion of reads mapping to mitochondrial genomes and cytosine to thymine misincorporation at the 5′ end of reads are shown across voucher preservation methods, with specimen age treated as either: (A) a categorical variable binned into young (1980–present) and old (1870–1965) age classes, or (B) as a continuous variable in linear regression models. Mitochondrial mapping was used to minimize reference genome divergence and assess the robustness of qualitative patterns observed using nuclear reference genomes.
**Figure S3:** Median fragment length of mapped sequencing reads estimated from nuclear and mitochondrial reference genome mapping. Median mapped read length is shown across voucher preservation methods and specimen age for reads mapped to nuclear reference genomes (top panel) and mitochondrial reference genomes (bottom panel), with specimen age treated as either: (A) a categorical variable binned into young (1980–present) and old (1870–1965) age classes, or (B) as a continuous variable in linear regression models. Patterns in mapped read length broadly mirror extract‐based fragment size estimates, supporting the robustness of fragmentation trends inferred from TapeStation profiles.
**Figure S4:** Effect of sequencing depth on measures of DNA quality and quantity. Endogenous content (post‐filter), median mapped read length, and 5′ cytosine‐to‐thymine misincorporation (mean of the first two positions) were recalculated following random downsampling of high‐coverage libraries to a range of input read depths (1000 reads to the full dataset). Points represent the mean across independent downsampling replicates, with error bars indicating ±1 standard deviation across independent random downsamplings.
**Figure S5:** Effect of tissue type on measures of DNA quality and quantity in alcohol‐fixed specimens across taxonomic groups: (A) DNA yield per unit mass of tissue, (B) DNA peak fragment size, (C) endogenous content, the fraction of sequenced reads mapping to the reference genome, and (D) misincorporation of cytosine at the 5′ end of reads. Specimens are further split by age group: young (1980‐present) or old (1870–1965).
**Figure S6:** Interaction of tissue type, taxonomic group, and age on DNA quality measures in alcohol‐fixed specimens. Post hoc interaction plots showing the estimated marginal means (±95% confidence intervals) for (1) DNA yield per mg of tissue, (2) DNA peak fragment size, (3) endogenous DNA content, and (4) misincorporation of cytosine at the 5′ end of reads, across four tissue types (liver, muscle, skin, and bone) and three vertebrate taxonomic groups (amphibians, reptiles, birds) from alcohol‐fixed specimens. Data are split into two age categories: young (1980–present) and old (1870–1965).
**Figure S7:** Effect of tissue type on measures of DNA quality and quantity in formalin‐fixed specimens across taxonomic groups: (A) DNA yield per unit mass of tissue, (B) DNA peak fragment size, (C) endogenous content, the fraction of sequenced reads mapping to the reference genome, and (D) misincorporation of cytosine at the 5′ end of reads. Specimens are further split by age group: young (1980‐present) or old (1870–1965).
**Figure S8:** Interaction of tissue type, taxonomic group, and age on DNA quality measures in formalin‐fixed specimens. Post hoc interaction plots showing the estimated marginal means (±95% confidence intervals) for (1) DNA yield per mg of tissue, (2) DNA peak fragment size, (3) endogenous DNA content, and (4) misincorporation of cytosine at the 5′ end of reads, across four tissue types (liver, muscle, skin, and bone) and three vertebrate taxonomic groups (amphibians, reptiles, birds) from formalin‐fixed specimens. Data are split into two age categories: young (1980–present) and old (1870–1965).
**Table S3:** Post hoc pairwise comparisons of linear regression slopes assessing the relationship between specimen age and four DNA quality metrics across preservation methods (dried skin, alcohol‐fixed, and formalin‐fixed specimens). Values indicate the estimated difference in slope between preservation types for each DNA metric: DNA yield (ng/mg), DNA fragment size (bp), endogenous content (%), and cytosine to thymine (C to T) deamination at the first base. A positive estimate indicates a steeper slope in the first group relative to the second. Significant values (*p* < 0.05) are bolded.

**Table S1:** Information on voucher specimens analysed, including preservation method, taxonomic information, year of collection, extraction and sequencing statistics (DNA quantity, fragment size, estimated endogenous content, and C to T deamination at the first 5′ base).
**Table S2:** Information on extraction statistics of different tissue types across the fluid‐preserved voucher specimens, including preservation method, taxonomic information, year of collection, extraction statistics (DNA quantity, fragment size, estimated endogenous content, and C to T deamination at the first 5′ base).

## Data Availability

Measures of DNA quality are available in the Supporting Information. Demultiplexed raw shotgun sequencing data have been uploaded to the European Nucleotide Archive (study accession PRJEB126598).
