# Supplementary material for: A Comparative Survey of Historical DNA Degradation From Vertebrate Museum Specimens: Unexpected Utility of Fluid‐Preserved Specimens
Source: Mol Ecol Resour. 2026 Oct 20;26(7):e70206. doi: 10.1111/1755-0998.70206 (PMC13682614; doi:10.1111/1755-0998.70206)
Supplement: Supplementary file 1 [file MEN-26-e70206-s002.docx]

Supplementary material for

**A comparative survey of historical DNA degradation profiles from dry, frozen, and fluid-preserved museum specimens**

Alexander T. Salis, Nasrin Ali, Shiva Karmakar, Jasleen Kaur, Brian Tilston Smith, Christopher J. Raxworthy

Corresponding author: A.T.S. (alexander.t.salis@gmail.com)

**This PDF file includes:**

Supplementary text

Tables S1 to S2

Figures S1 to S4

**Supplementary Information Text**

**Extended Material and Methods**

***DNA extraction from voucher specimens***

Tissue subsamples from all voucher specimens were weighed, minced with a razor blade, and then washed quickly with 70% ethanol followed by washing twice with ddH_2_O for approximately two hours each time. Whole genomic DNA was then extracted using a modified protocol using a Qiagen™ DNeasy Blood & Tissue kit with QIAquick spin columns. The same, degraded DNA-optimized, extraction protocol was used for all voucher specimens (i.e., specialized formalin-fixed protocols were not considered) to allow direct comparisons of DNA quantity and quality measures. Firstly, tissue was digested in 180uL of buffer ATL with 30 uL of proteinase K overnight at 56 °C on a thermomixer for at least 16 hrs. Following digestion, 200 uL of buffer AL was added and sample vortex before adding 250uL of 100% ethanol and vortexing. The sample mix was then transferred to a QIAquick spin column and spun at 5,000 g. QIAquick columns were used instead of the provided DNeasy columns due to the ability to bind smaller DNA fragments. The DNA was then washed with 500 uL of AW1, spun again at 5,000 g, and then washed with 500uL of AW2 and spun at maximum speed for 3 minutes. This was followed by a dry-spin at maximum speed for 1 min. DNA was then eluted in 100uL of TE buffer (10 mM Tris-HCl and 1 mM EDTA), spun at 5,000 g for 1 min to collect in a clean 1.5mL Eppendorf. A second elution in 100ul TE was performed to maximize DNA recovery and collected into the same Eppendorf tube.

***DNA Library Preparation and Sequencing***

To minimize library preparation method biases, all DNA extracts, modern and historical, were subjected to the same library preparation method. First, modern extracts and historical extracts with large fragment sizes (>1000 bp peak fragment size) were concentrated to approximately 50 uL using an Eppendorf 5301 Vacufuge Concentrator System (Hamburg, Germany) and then fragmented using a Covaris® ME220 (Covaris, LLC, Woburn, MA) machine, using 4 microTUBE-50 screw-cap PN 520166. DNA libraries from all DNA extracts were then constructed using the BEST protocol (Carøe et al., 2018) with modifications as per Mak et al. (2017). Library input DNA was not normalised by concentration, instead, a fixed volume (16 µL) of extract was used for all libraries. As a result, absolute DNA input varied among samples depending on extract concentration. Briefly, 16 µL of extracted DNA was combined with 0.2 uL T4 DNA polymerase (3 U/μL), 0.6 uL T4 PNK (10 U/μL), 2 μL 10x T4 DNA Ligase Buffer, 0.2 μL dNTP (25 mM) and 1 μL of a reaction enhancer (containing 25% PEG-4000, 2 μg/μL BSA and 400 mM NaCl). The reaction was then incubated at 20 °C for 30 minutes followed by 65 °C for 30 minutes. Ligation of adapter sequences was performed by the addition of 1 μL of BEDC3 adapter sequence (20 mM stock) and mixed followed by the addition of 3 μL of 50% PEG 4000, 0.5 μL T4 DNA Ligase Buffer (10x) and 0.5 μL T4 DNA ligase (400 U/μL). The reaction was then gently mixed by pipette mixing and incubated at 20 °C for 30 minutes followed by 10 minutes at 65 °C. Fill in reactions were then performed by adding 0.2 μL dNTP (25 mM), 1 μL 10x Isothermal Amplification Buffer 10x, 0.8 μL Bst 2.0 Warmstart Polymerase (8 U/uL), and 3 μL molecular biology grade water. The final reaction was then incubated at 65 °C for 20 minutes followed by 80 °C for 20 minutes.

Following preparation of libraries, DNA was cleaned using a Qiagen MineElute Reaction Cleanup Kit following manufacturer's instructions and eluting in 22.5 μL EB buffer with 0.05% Tween 20. Double-indexed Illumina adapters were added to DNA libraries through a round of indexing PCRs using PCR primers complementary to the BEDC3 adapter. P5 and P7 adapter sequences contained barcode sequences to allow multiplexing during subsequent high-throughput sequencing. Two technical replicates of each PCR of a library were performed to minimize PCR bias and maintain library complexity. Each PCR totaled 50 μL and contained 1× Gold buffer, 2.5 mM MgCl2, 1 mM dNTPs, 0.5 mM each indexing primer, 0.1 U AmpliTaq Gold DNA Polymerase and 6 μL of library DNA. Cycling conditions were 94 °C for 12 min, 20 cycles of 94 °C for 30 s, 60 °C for 30 s, and 72 °C for 45 s, followed by 72 °C for 10 min. PCR replicates were then pooled for each library and cleaned using AxyPrep™ magnetic beads (Axygen™) following manufacturers protocols and eluting in 30 μL of H2O and quantified with a Qubit fluorometer using a Qubit dsDNA high sensitivity assay kit. DNA libraries were pooled and sent for shotgun 150 bp paired-end sequencing on an Illumina HiSeq X Ten or NovaSeq 6000 at Novogene Co or Azenta/GENEWIZ, aiming for at least 100,000 read pair sequencing depth per library.

**Table S1:** Information on voucher specimens analysed, including preservation method, taxonomic information, year of collection, extraction and sequencing statistics (DNA quantity, fragment size, estimated endogenous content, and C to T deamination at the first 5’ base).

See Excel File

**Table S2:** Information on extraction statistics of different tissue types across the fluid-preserved voucher specimens, including preservation method, taxonomic information, year of collection, extraction statistics (DNA quantity, fragment size, estimated endogenous content, and C to T deamination at the first 5’ base).

See Excel File

**Table S3:** Post-hoc pairwise comparisons of linear regression slopes assessing the relationship between specimen age and four DNA quality metrics across preservation methods (dried skin, alcohol-fixed, and formalin-fixed specimens). Values indicate the estimated difference in slope between preservation types for each DNA metric: DNA yield (ng/mg), DNA fragment size (bp), endogenous content (%), and cytosine to thymine (C to T) deamination at the first base. A positive estimate indicates a steeper slope in the first group relative to the second. Significant values (p < 0.05) are bolded.

| **Contrast** | **Estimate of difference in slope** | **SE** | **df** | **t.ratio** | **p.value** |
| --- | --- | --- | --- | --- | --- |
| **DNA quantity** | | | | | |
| **Alcohol - Formalin** | 1.66 | 0.677 | 113 | 2.47 | **0.0392** |
| **Alcohol - Skin** | -2.71 | 0.665 | 113 | -4.07 | **0.0002** |
| **Formalin - Skin** | -4.37 | 0.665 | 113 | -6.563 | **<.0001** |
| **Fragment length** | | | | | |
| **Alcohol - Formalin** | 15.1 | 8.75 | 112 | 1.727 | 0.1965 |
| **Alcohol - Skin** | -30.4 | 8.6 | 112 | -3.533 | **0.0016** |
| **Formalin - Skin** | -45.5 | 8.5 | 112 | -5.352 | **<.0001** |
| **Endogenous DNA** | | | | | |
| **Alcohol - Formalin** | 0.1192 | 0.111 | 113 | 1.076 | 0.5307 |
| **Alcohol - Skin** | -0.0036 | 0.108 | 113 | -0.033 | 0.9994 |
| **Formalin - Skin** | -0.1227 | 0.108 | 113 | -1.134 | 0.4953 |
| **C to T deamination** | | | | | |
| **Alcohol - Formalin** | 3.66e-06 | 0.000103 | 113 | 0.035 | 0.9993 |
| **Alcohol - Skin** | -1.64e-04 | 0.000101 | 113 | -1.621 | 0.2410 |
| **Formalin - Skin** | -1.68e-04 | 0.000101 | 113 | -1.657 | 0.2262 |

**Figure S1:** Effect of museum voucher preservation method on endogenous content and cytosine deamination when analyses are restricted to specimens with close nuclear reference genomes. Endogenous content (fraction of sequenced reads mapping to the nuclear reference genome) and cytosine to thymine misincorporation at the 5′ end of reads are shown with specimen age treated as either: A) a categorical variable binned into young (1980–present) and old (1870–1965) age classes, or B) as a continuous variable in linear regression models. Restricting analyses to specimens with close reference genomes does not qualitatively alter patterns among voucher types observed in the full dataset.

**Figure S2:** Endogenous content and cytosine deamination estimated from reads mapped to mitochondrial reference genomes. The proportion of reads mapping to mitochondrial genomes and cytosine to thymine misincorporation at the 5′ end of reads are shown across voucher preservation methods, with specimen age treated as either: A) a categorical variable binned into young (1980–present) and old (1870–1965) age classes, or B) as a continuous variable in linear regression models. Mitochondrial mapping was used to minimise reference genome divergence and assess the robustness of qualitative patterns observed using nuclear reference genomes.

**Figure S3:** Median fragment length of mapped sequencing reads estimated from nuclear and mitochondrial reference genome mapping. Median mapped read length is shown across voucher preservation methods and specimen age for reads mapped to nuclear reference genomes (top panel) and mitochondrial reference genomes (bottom panel), with specimen age treated as either: A) a categorical variable binned into young (1980–present) and old (1870–1965) age classes, or B) as a continuous variable in linear regression models. Patterns in mapped read length broadly mirror extract-based fragment size estimates, supporting the robustness of fragmentation trends inferred from TapeStation profiles.

**Figure S4:** Effect of sequencing depth on measures of DNA quality and quantity. Endogenous content (post-filter), median mapped read length, and 5′ cytosine-to-thymine misincorporation (mean of the first two positions) were recalculated following random downsampling of high-coverage libraries to a range of input read depths (1,000 reads to the full dataset). Points represent the mean across independent downsampling replicates, with error bars indicating ±1 standard deviation across independent random downsamplings.

**Figure S5:** Effect of tissue type on measures of DNA quality and quantity in alcohol-fixed specimens across taxonomic groups: A) DNA yield per unit mass of tissue, B) DNA peak fragment size, C) endogenous content, the fraction of sequenced reads mapping to the reference genome, and D) misincorporation of cytosine at the 5’ end of reads. Specimens are further split by age group: young (1980-present) or old (1870-1965).

**Figure S6:** Interaction of tissue type, taxonomic group, and age on DNA quality measures in alcohol-fixed specimens. Post-hoc interaction plots showing the estimated marginal means (±95% confidence intervals) for 1) DNA yield per mg of tissue, 2) DNA peak fragment size, 3) endogenous DNA content, and 4) misincorporation of cytosine at the 5’ end of reads, across four tissue types (liver, muscle, skin, and bone) and three vertebrate taxonomic groups (amphibians, reptiles, birds) from alcohol-fixed specimens. Data are split into two age categories: young (1980–present) and old (1870–1965).

**Figure S7:** Effect of tissue type on measures of DNA quality and quantity in formalin-fixed specimens across taxonomic groups: A) DNA yield per unit mass of tissue, B) DNA peak fragment size, C) endogenous content, the fraction of sequenced reads mapping to the reference genome, and D) misincorporation of cytosine at the 5’ end of reads. Specimens are further split by age group: young (1980-present) or old (1870-1965).

**Figure S8:** Interaction of tissue type, taxonomic group, and age on DNA quality measures in formalin-fixed specimens. Post-hoc interaction plots showing the estimated marginal means (±95% confidence intervals) for 1) DNA yield per mg of tissue, 2) DNA peak fragment size, 3) endogenous DNA content, and 4) misincorporation of cytosine at the 5’ end of reads, across four tissue types (liver, muscle, skin, and bone) and three vertebrate taxonomic groups (amphibians, reptiles, birds) from alcohol-fixed specimens. Data are split into two age categories: young (1980–present) and old (1870–1965).
